# Tibialis Anterior Muscle Hernia in a Young Woman

**DOI:** 10.7759/cureus.36596

**Published:** 2023-03-23

**Authors:** Abdullah M AlZahrani, Felwa A AlMarshad, Nehal A Mahabbat, Nora N Alsaud, Khalid Fayi, Yazeed A Jarman, Raghad AlKhashan, Abdulaziz Jarman

**Affiliations:** 1 Plastic and Reconstructive Surgery, King Faisal Specialist Hospital and Research Centre, Riyadh, SAU; 2 Medicine, King Saud Bin Abdulaziz University for Health Sciences, Riyadh, SAU; 3 Medicine, King Saud University, Riyadh, SAU

**Keywords:** ortho surgery, plastic and reconstructive surgery, myofascial defect, myofascial herniation, tibialis anterior herniation

## Abstract

Muscle herniation is defined as a myofascial defect resulting in protruding of the muscle through the fascia covering it. It can present anywhere in the body, the most common is the lower limbs. Tibialis muscle herniation is considered a rare entity with few reported cases. Here, we present the case of a 24-year-old Saudi female patient who complained of swelling and pain in the anterior aspect of the left leg for three months. She underwent surgical repair of the fascia with a good outcome.

This case presentation aims to contribute to the literature on myofascial herniation by specifically addressing tibialis anterior herniation of the leg and emphasizing the importance of considering it a differential diagnosis in similar presentations. This report highlights the excellent surgical outcomes and satisfactory results in patients with muscle herniation.

## Introduction

Muscle herniation is a myofascial defect in which the muscle tissue protrudes past the fascia it is invested in. Only around 200 reported cases exist in the literature concerning muscle herniations within the extremities most typically involving the tibialis and forearm muscles [1], thus making it a rare finding. This rarity in turn leads to this condition being overlooked and misdiagnosed as a more sinister pathology such as lipoma, hematoma, or a soft tissue neoplasm. Therefore, in the absence of alarming findings in the history and physical exam, the practitioner should avoid undertaking unnecessary investigations, as it can be an undue burden upon the patient or the healthcare system. These herniations are usually asymptomatic and present as subcutaneous nodules of the lower extremity that change with limb position [2-4]. They appear to be more common in men; however, there are recent case reports in women and in the adolescent population [1,4].

## Case presentation

**Figure 1:**
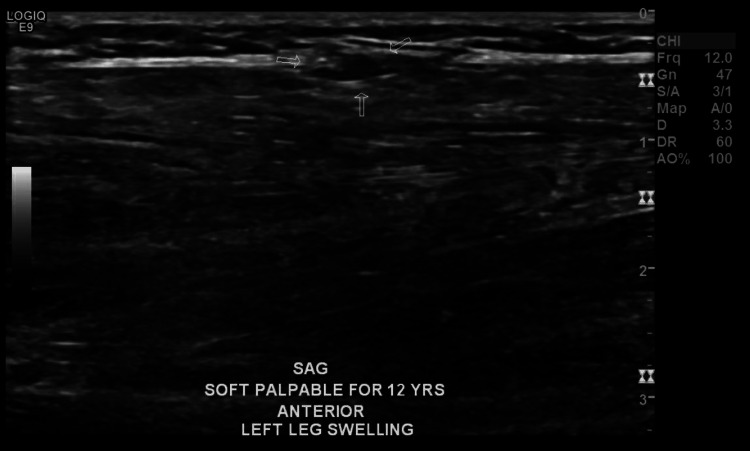
Ultrasound revealing the herniated muscle (arrows)

A 24-year-old healthy female, presented to the clinic with anterior left leg swelling that had been persistent for three months. It was not progressing and was associated with pain upon exertion. There was no history of growth or skin changes and history of trauma. Upon examination, there was a well-circumscribed swelling on the anterior left leg, approximately 1 cm x 1 cm, non-tender, and was more prominent in the fencer's lunge position. Dynamic ultrasonography and MRI were ordered for investigation. The MRI showed no abnormal findings. Dynamic US showed a muscular facial defect at the anterior aspect of the mid-left leg (at the area of complaint), measuring around 1.3 cm and associated with muscle fiber herniation which is exaggerated with compression/ The overall findings were compatible with muscle herniation (Figure 1). No gross soft tissue lesion was seen, nor was there abnormal vascularity on Doppler images.

**Figure 2:**
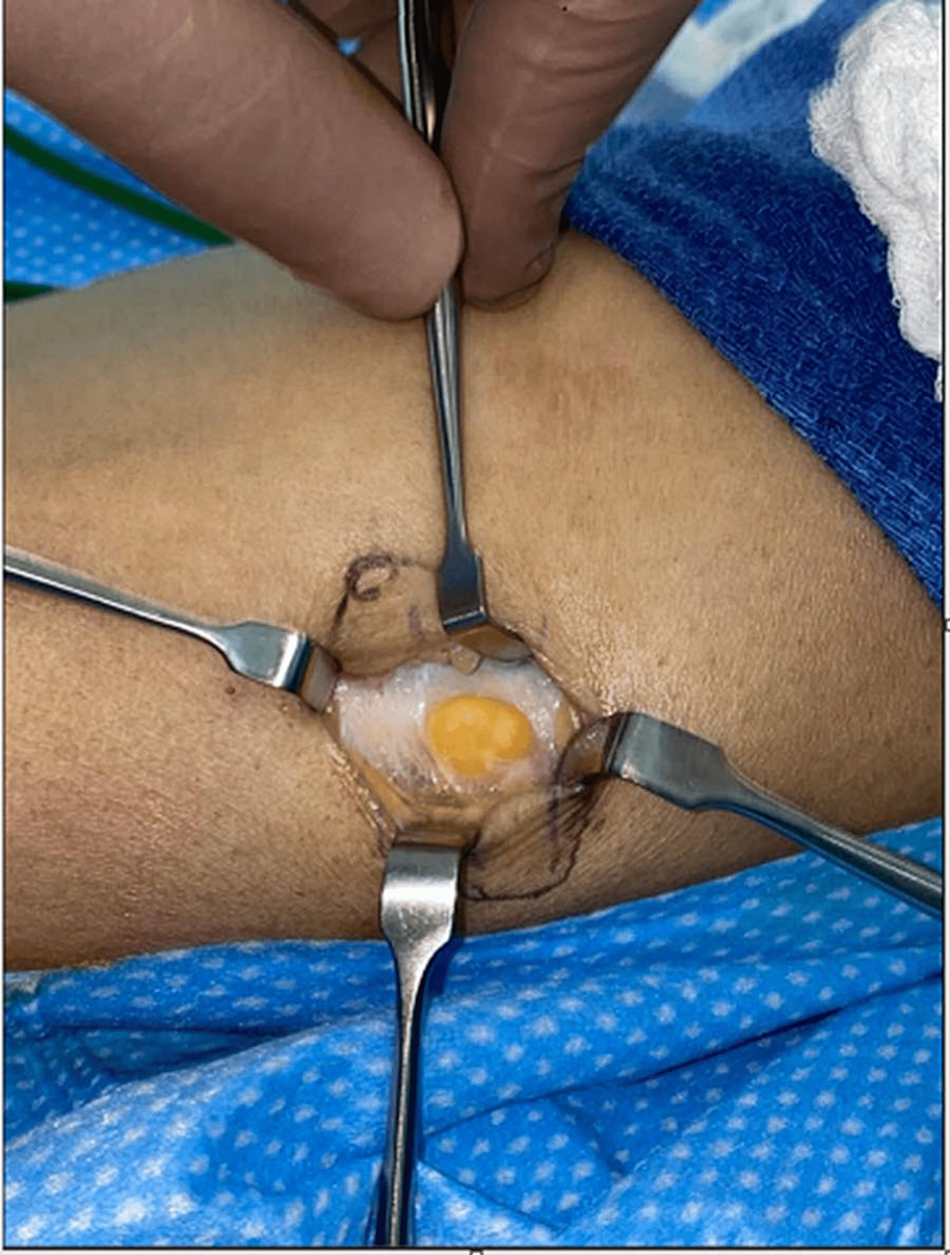
Fascia defect with herniated fat

**Figure 3:**
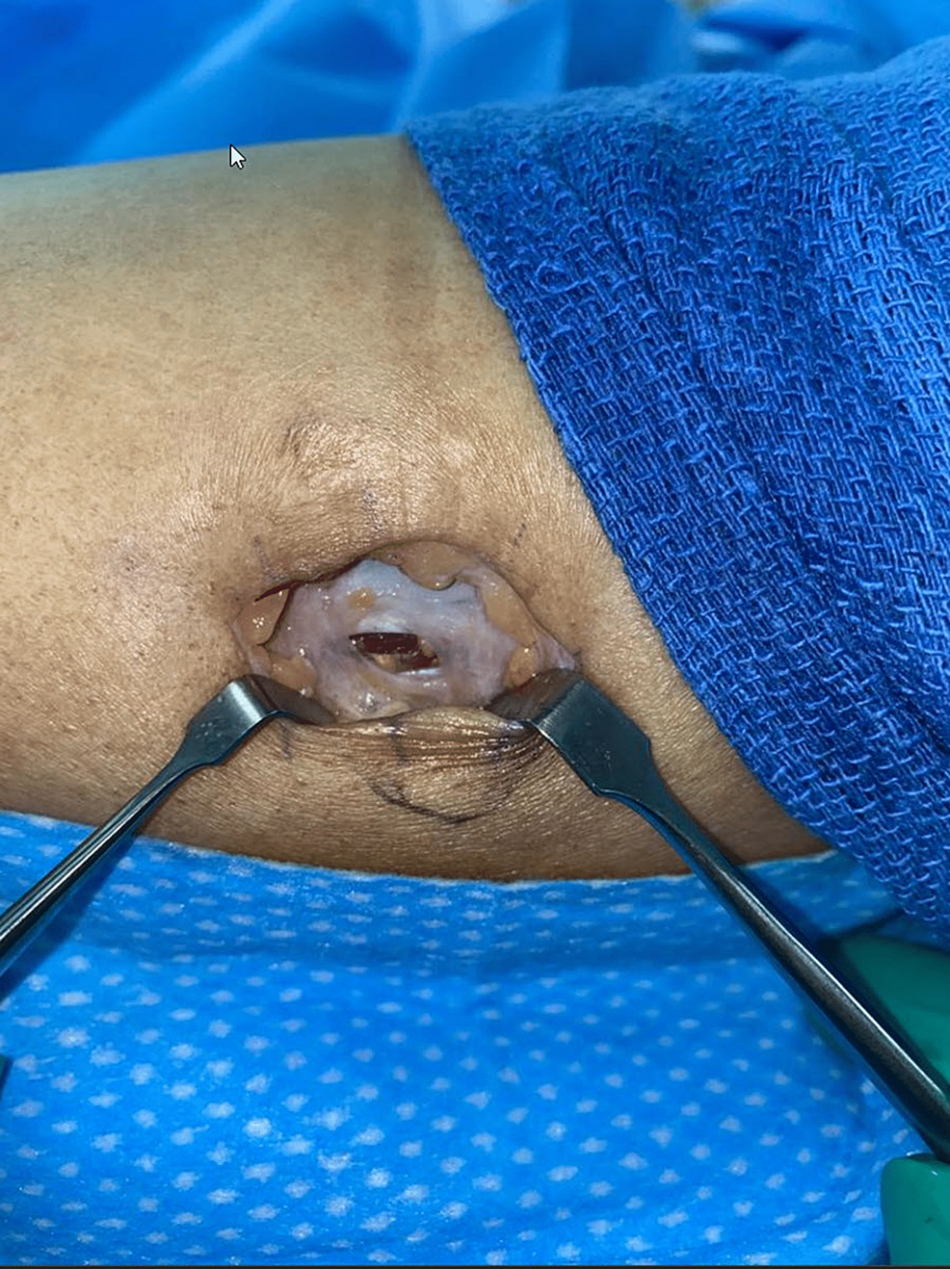
Fascia defect on the tibialis anterior muscle

**Figure 4:**
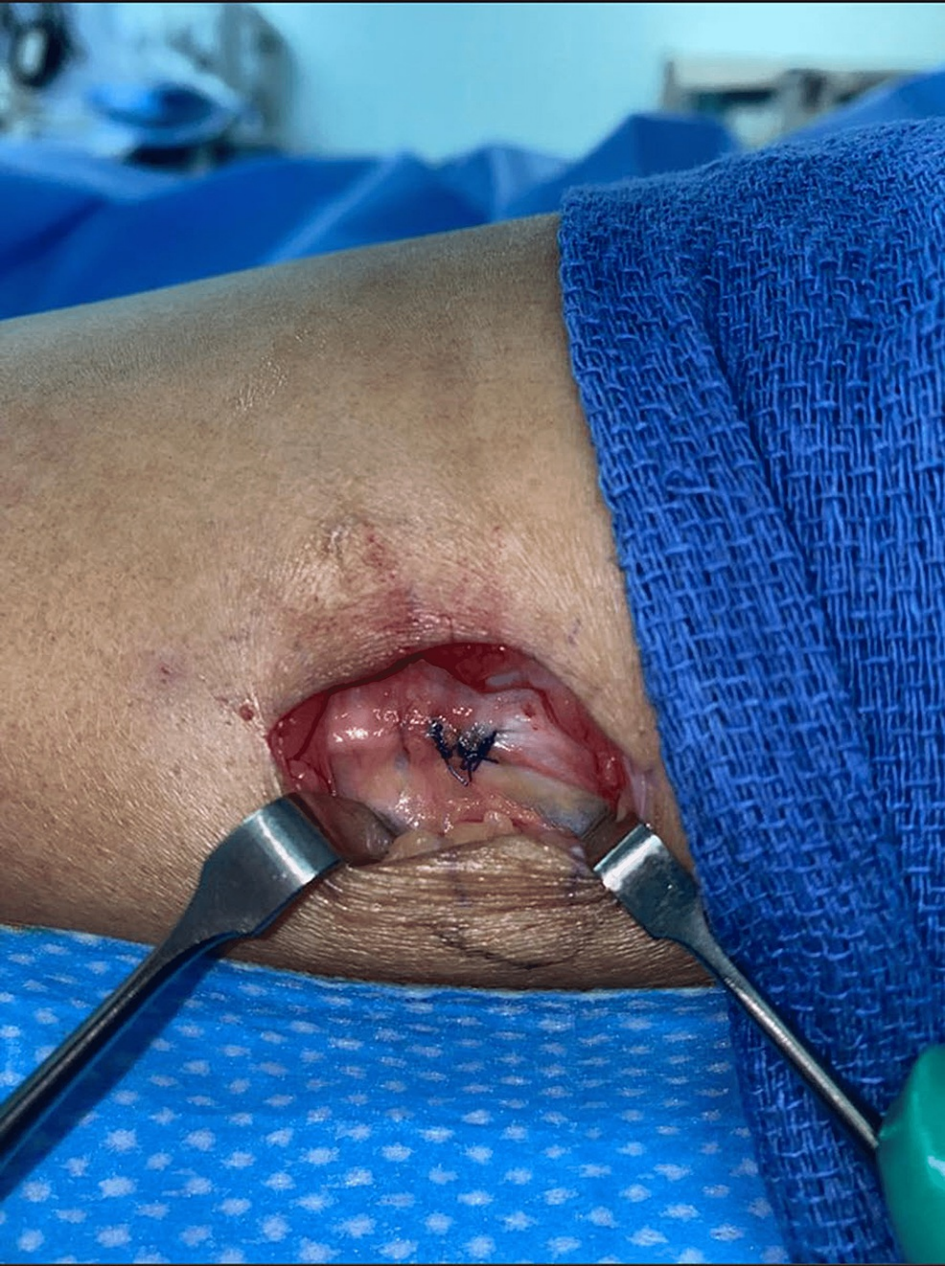
The fascia after the repair

This patient was initially managed conservatively for five months with regular follow-ups. Despite this, the patient remained symptomatic, after which surgical exploration was offered to the patient. Surgical exploration was done with a lazy S incision over the swelling. During the exploration, the fascial defect was identified by palpation. The herniation matter was visible and was found to be subcutaneous fat with an underlying defect on the tibialis anterior muscle (Figures 2, 3). It was roughly 1 cm in diameter. The fascia was repaired by polydioxanone (PDS) 3-0 figure-of-eight and reinforced by continuous suture (Figure 4). The skin was sutured with Monocryl 3-0 and then 3-0 subcuticular. At the follow-up four months post-surgery, the patient had a healed wound and was without pain and bulging of the previous mass.

## Discussion

Anterior tibialis hernia was first described in a case series conducted by the Swedish surgeon Ihde back in 1929 in which he classified lower limb hernias into two categories, acquired (post-traumatic); because of a defect in the deep myofascial fascial secondary to trauma, or constitutional; caused by weakness of muscular fascia or arising at sites of focal fascial weakness [3]. Muscle hernias commonly involve the lower extremities, particularly the anterolateral compartment of the leg. Due to its superficial and tight fascia, these include tibialis anterior hernias. They are usually reducible but can be irreducible when the muscle is strangulated in the hernia [2-5]. Muscle hernias are typically asymptomatic. Yet some patients may complain about pain, weakness, tenderness, cramping, or change in sensation [4].

On physical examination, provocative maneuvers can be useful in diagnosing tibialis anterior hernias such as dorsiflexion of the foot [4]. Differential diagnoses include lipoma, varicosities, focal rupture of muscle presenting as pseudohernia, and arteriovenous malformation [4]. Complications of muscle herniation may be linked to pain, abnormal sensation, and compartment syndrome [2]. A detailed history and physical examination, along with investigations are crucial to diagnosing tibialis anterior hernia. Several modalities can be utilized to diagnose tibialis anterior herniation, such as dynamic ultrasonography that can show typical myofascial defect as diagnostic of focal muscle herniation. This can help avoid further imaging and unnecessary invasive procedures while providing assurance to the patient simultaneously [2]. Previously, the diagnosis was made on the basis of a focal swelling or nodule that can enlarge or reduce in size with contraction [6], but currently, dynamic ultrasonography is the gold standard [7-8]. Another modality that is more favorable for preoperative evaluation is MRI that can be used to assess the defect and surrounding soft tissue structures [5]. Occasionally, Doppler imaging shows vessels at the site of herniation. This observation encourages the theory that hernias can occur at areas of fascial weakness, such as the entering sites of perforating vessels, and must not be mistaken with a sign in favor of a tumor mass [3].

There has been controversy regarding the treatment of tibialis anterior herniation, however, in the literature, asymptomatic hernias are preferably treated conservatively. Conservative options include rest, exercise restriction, and compression stockings. Surgical options that have been described in the literature include fasciotomy, primary repair of fascia, mesh repair, tibial periosteal flap, and fascia graft of the tensor fascia lata. There has been a report of a case that developed compartment syndrome with primary closure, however, with this approach, the results are more cosmetically appealing, thus it was suggested that small defects can be repaired with this method, and large defects have a better outcome with fasciotomy [8-9].

## Conclusions

The purpose of this report is to raise awareness among practitioners about muscle herniations in order to not confuse them with sinister pathologies and place a burden of costs and distress. We recommend further investigations in determining which treatment option is superior to better aid the management of these muscle herniations.
